# Laser Therapy for Onychomycosis: Fact or Fiction?

**DOI:** 10.3390/jof1010044

**Published:** 2015-04-03

**Authors:** Lucette Teel Liddell, Ted Rosen

**Affiliations:** Baylor College of Medicine, Department of Dermatology, 1977 Butler Blvd, Suite E6.200, Houston, TX 77030, USA; E-Mail: Lucette.Liddell@bcm.edu

**Keywords:** laser, laser therapy, onychomycosis

## Abstract

Onychomycosis is a common fungal infection, afflicting some 10% of the adult population in industrialized countries. Aside from cosmetic concerns, onychomycosis can be the cause of toe and foot pain, as well as the underlying etiology for serious secondary bacterial infections and traumatic ulcerations. In select populations, such as diabetics, the latter conditions may even result in loss of all or part of the lower extremity. Thus, a simple, cost-effective and safe treatment for onychomycosis is highly desirable. Although both topical and oral systemic antifungal agents are available for this purpose, they are not always effective, carry some medical risks, are associated with potentially significant drug-drug interactions, and may be unacceptable to patient and healthcare provider alike. Physical modalities, such as laser therapy, therefore appear appealing. The question is whether laser treatment is sufficiently efficacious and safe to warrant the current high cost per treatment. The readily available literature on this controversy will be reviewed herein.

## 1. Introduction

Onychomycosis is a fungal infection of the nail that poses a significant treatment challenge, as it is often completely or partially refractory to approved topical and systemic medications. Estimated to affect 2%–10% of the population, onychomycosis is the most common pathology of the nail, comprising 18%–40% of all nail disorders [1,2]. The predominant nail pathogens are dermatophytes *Trichophyton rubrum* and *Trichophyton mentagrophytes*, though yeasts and non-dermatophyte molds may also be etiologic, particularly in diabetic and immunosuppressed patients. Fungi colonize the nail plate, bed, and matrix, leading to discoloration of the nail, thickening of the subungual region, and, when advanced, onycholysis [3,4]. Beyond cosmetic concern, onychomycosis may lead to secondary bacterial infections and predispose to erysipelas and cellulitis [5]. In the diabetic population, jagged onychomycotic toenails may injure the adjacent tissue and thus increase the risk of foot ulcer, with sepsis or amputation a potential consequence [6].

Systemic antifungal drugs are the mainstay of therapy for this chronic disease. When taken for a 12-week course, oral terbinafine has a mycologic cure rate of 71%–82% and a maximal clinical response rate of 60%–70% [7,8]. However, patients are often poorly compliant with the lengthy duration of treatment, resulting in sub-therapeutic concentrations reaching the nail plate [9]. Additionally, terbinafine can elevate liver enzymes and has rarely progressed to fulminant liver failure. Accordingly, it requires routine blood testing and is contraindicated in patients with chronic or active liver disease [10]. Additional adverse events include headache, loss of taste, and abdominal discomfort and induction of a lupus-like syndrome. Itraconazole, another FDA-approved systemic antifungal drug utilized to manage onychomycosis, has much lower mycologic and clinical clearing rates, and may induce cardiac toxicity; it is also associated with a plethora of drug-drug interactions, making it an equally unattractive treatment modality.

In light of these limitations, laser therapy has been proposed as an alternative option for onychomycosis therapy. Currently utilized for a wide variety of medical and cosmetic skin disorders, dermatological lasers, supporters argue, offer a convenient solution with minimal side effects [11]. Treatment is administered in the medical setting (office/hospital), thus eliminating the requirement for patient adherence. Laser therapy also has the potential to treat those patients where systemic antifungals are either contraindicated or associated with possible drug-drug interactions [4].

Although the mechanism of action remains unknown, several theories exist. By the principle of selective photothermolysis, based on differences in thermal conductivity, laser energy may be preferentially absorbed by fungal pathogens resulting in photothermal and photomechanical damage that spares surrounding human tissues [4,12]. An alternate theory suggests the formation of free radicals by incident laser energy and light absorption by fungal pigment xanthomegnin, present in high concentration in *Trichophyton rubrum* [13].

Since 2010, eight lasers have received regulatory approval for the “temporary cosmetic improvement” of onychomycosis, the clearance of these devices established upon “substantial equivalence” in technological specifications and safety to predicate devices [3,14]. Thus far, laser device systems have not been approved as a fungicidal therapy for onychomycosis, unlike, for example, terbinafine and azole drugs whose direct antimycotic mechanism is known. This paper seeks to review the existing literature evaluating laser efficacy in onychomycosis with regard to clearance of pathogen, as well as improvement in nail appearance.

## 2. Methods

We conducted an extensive PubMed literature search, with the following criteria: “(laser) AND (onychomycosis)” and “(onychomycosis) AND (laser)”. Articles written in a language other than English or prior to ten years ago were excluded. Of the 70 eligible results, 17 were original studies, examined presently, 21 were reviews, and 32 were other commentaries or correspondence (Table S1).

## 3. Results and Discussion

### 3.1. Results

#### 3.1.1. Short Pulse Nd:YAG Lasers

##### *In Vivo* Studies

Solid state lasers, such as the neodymium-doped yttrium aluminum garnet (Nd:YAG) laser, constitute the largest class of FDA-approved devices for onychomycosis. They are designed to deliver energy continuously or in pulses of milliseconds, microseconds, nanoseconds, or femtoseconds, the energy density increasing with shorter pulse durations [3].

In 2011, Hochman designed a pilot study of eight patients with culture- or PAS-confirmed onychomycosis to be treated with 2–3 treatments of a 0.65-ms pulsed LightPod Neo Nd:YAG laser [15]. The patients were encouraged to use an antifungal cream daily during the treatment period. Seven patients had negative post-treatment cultures when reassessed at four months after the final treatment. The study was limited by small sample size and potentially confounded by the use of topical antifungal. The degree of clinical improvement in the nails was not quantified.

Following Hochman’s pilot study, Kimura and colleagues enrolled 13 patients (37 toenails) to be treated with a 0.3-ms Nd:YAG laser [16]. Subjects received one to three treatments at four or eight week intervals, for an average of 2.4 treatments per patient. To measure clinical improvement, the authors calculated the ratio of clear nail growth to total length of toenail at baseline and at 8, 16, or 24 weeks post-treatment. They found 30 toenails (81%) with “complete” or “moderate” to “significant” clearance. Nineteen of those toenails (51%) showed “complete” clearance of which 100% tested negative for fungi on direct microscopy. This study was limited by small sample size and the authors did not stratify improvement by patient. In addition, it was unclear what degree of change in the nail merited designation of “moderate” or “significant” clearance. Noted in the authors’ disclosure, an equipment loan was received from laser production company, Cutera, Inc. (Bayshore Blvd., Brisbane, CA, USA).

Waibel assigned 21 patients with onychomycosis verified by culture and PAS stain into three treatment arms, corresponding to a 1064-nm laser, 1319-nm laser, or BroadBand Light from a filtered flash lamp [17]. Patients received four treatments one week apart. At six months, 20 of 21 patients had negative cultures. Though clinical improvement was not quantified, in general, toenails had decreased subungual debris, discoloration, and onycholysis. One of the authors of the study disclosed a financial interest in the commercialization of this laser technology.

S. Tyler Hollmig and colleagues conducted the first randomized clinical trial evaluating FDA-approved JOULE ClearSense Nd:YAG laser [18]. The authors randomized 27 patients with culture-confirmed toenail onychomycosis in a 2:1 ratio of treatment group to control group. Using the protocol recommended by Sciton, patients received two sessions separated by a two-week interval. At three months, the authors found no statistically significant difference between treatment and control groups when evaluating negative culture in all ten toenails (*p* = 0.49) and proximal nail plate clearance (*p* = 0.18). At 12 months, the modest improvement of proximal nail plate clearance seen in the laser group was not sustained when compared to the control (0.24 mm *vs.* 0.15 mm, *p* = 0.59).

##### *In Vitro* Studies

In a controlled pilot study, Choi investigated a 1444-nm Nd:YAG laser with toenail scrapings from 20 patients with onychomycosis [19]. The trial consisted of three arms: a control group and two treatment groups at 300 and 450 J. Compared to the control, the authors found an average reduction rate in the number of colony-forming units (CFUs) of 75.9% for the group treated with 300 J and 85.5% for those receiving 450 J, with no significant difference between the two energy settings. At 450 J, scanning electron microscopy revealed disintegration of fungal and toenail structures in the nail plate, and the dishes displayed “marked disfigurements”. The authors admitted that “among the controls, the number of CFUs was highly variable”.

Carney and colleagues conducted a multi-part study to determine the fungicidal temperature of *Trichophyton rubrum in vitro* and whether it is reproducible by a Nd:YAG 1064-nm laser *in vivo* [20]. They found a fungicidal effect for *T. rubrum* at 50 °C after 15 min. Fungicidal effect was determined by counting the number of colony-forming units on potato dextrose agar after exposure to seven different heat and time regimens: 5 min at 45 °C; 2, 5, 10, and 15 min at 50 °C; and 2 and 5 min at 55 °C. Confluent growth was defined as 400 CFU for *T. rubrum*. However, no growth inhibition was producible *in vitro* with direct laser irradiation to fungal colonies. The *in vitro* arms of the study were controlled and the examiners measuring colony growth were blinded. The *in vivo* portion of the study enrolled 10 patients with 14 onychomycotic great toenails for a 24-week pilot study, and treated them at a fluence of 16 J/cm^2^ and pulse duration 0.3 ms at weeks 0, 1, 2, 3, and 7. No control arm was used. To quantify clinical improvement, the authors calculated an Onychomycosis Severity Index (OSI) accounting for area of nail plate involvement, proximity of disease to the matrix, and degree of subungual hyperkeratosis [21]. Eight of the 14 nails showed improvement in the percent of disease involvement of the target nail, with two approaching more severe infection post-treatment. However, this improvement did not correlate with mycological cure assessed by culture and KOH. Moreover, the authors determined that the settings of the laser permitted a maximum temperature of 40 °C. Given patients’ complaints of mild pain at a fluence of 16 J/cm^2^, they concluded that aggressive settings to achieve the temperature necessary to effect cell death (50 °C) would not be practical clinically.

#### 3.1.2. Long Pulse Nd:YAG Lasers

In 2012, Zhang conducted a prospective clinical trial evaluating the 1064-nm PinPointe FootLaser with a 30-ms pulse duration [22]. Thirty-three patients (154 nails) were randomly distributed into two groups to receive four or eight sessions of laser treatment (groups 1 and 2, respectively), at one-week intervals. The patients were selected based on microscopic examination demonstrating fungal infection and followed at 8, 16, and 24 weeks post-treatment. To quantify clinical improvement, the authors calculated an “effective rate” representative of the percentage of newly grown nail with respect to baseline. In group 1, the effective rate at weeks 8, 16, and 24 was 63%, 62%, and 51%, respectively; in group 2, 68%, 67%, and 53%. The treatment effect was not significantly different between groups 1 and 2 (*p* > 0.05).The positive rates for microscopic examination and fungal culture were higher at 24 weeks than those at 8 weeks, suggesting relatively rapid recurrence of infection.

Moon and colleagues investigated the 1064-nm long-pulsed Sciton ClearSense laser of 0.3-ms pulse duration [23]. Thirteen patients (43 nails) with onychomycosis confirmed by KOH preparation and culture received five treatment sessions at 4-week intervals with a single follow-up at one month after the final treatment. They concluded that all patients achieved a “good response”, defined by least 50% clearance of turbid nail, however did not clarify as to whether this occurred in at least one nail or all affected nails. “Complete cure”, with negative culture and grossly normal appearing nail, was determined in four of the 43 nails (9.4%). The authors did not include patients who had total dystrophic onychomycosis. This study was limited by small sample size and lack of long-term data.

In a small study enrolling 12 patients with distal lateral subungual onychomycosis, Noguchi *et al.* [24] evaluated a 0.5-ms Nd:YAG laser. The patients received three treatments directed at a single great toenail and were evaluated at six months based on the reduction of turbidity observed in the nail plate. The authors reported six patients (50%) with lack of improvement or with worsening of the infection after treatment. In three cases the nail was cleared by an area greater than 70%. Visible cure was achieved in these three patients after six additional treatments; however, the authors did not confirm eradication of the pathogen. Patients with nail turbidity affecting >75% of the nail surface area were excluded from the study.

In a trial comparing long- and short-pulsed lasers, Hees treated the right and left great toenails of 10 patients separately with the PinPointe Footlaser (pulse duration 100 microseconds) and the Elite laser (pulse duration 40 ms), respectively [25]. Patients were also instructed to apply topical ciclopirox daily. Clinical improvement was indicated by a reduction in OSI score calculated by two blinded investigators. Mean OSI values diminished significantly from baseline by 3.8 points (15%; *p* = 0.006), 4.8 points (19%; *p* = 0.0002), and 2.9 points (12%; *p* = 0.04) over the course of three, six, and nine months, respectively. Lowest OSI values were reached at six months. They found no significant differences in the clearance of nail achieved by each laser. Contrasting their results to prior studies by Hochman and Kimura, discussed above, the authors concluded that lasers have only a temporary effect in onychomycosis and may require physical debridement of the nail as well as consistent laser treatments to maintain any curative effect [15,16]. This study was limited by the lack of topical ciclopirox only control arm to isolate the effect of the laser itself.

#### 3.1.3. Q-switched Lasers

The Light Age Q-Clear has a pulse duration in nanoseconds, achieving the highest energy density per pulse of all the Nd:YAG lasers [13]. In a study comparing Q-switched and long-pulsed laser systems, Hees exposed fungal colonies grown on agar plates to five different laser regimens previously reported to have an inhibitory effect on Trichophyton rubrum isolates *in vitro* [26,27,28]. Each plate was irradiated on one half with one of the following treatment regimens: 1064 nm Q-switched Nd:YAG laser at 4 and 8 J/cm^2^; 532 nm Q-switched Nd:YAG laser at 8 J/cm^2^; and 1064 nm long-pulsed Nd:YAG laser at 45 and 100 J/cm^2^. After six days, none of the fungal colonies showed signs of regression, nor were there significant differences between the parameters used or between treated and untreated colonies on the same plate (*p* ≥ 0.13). When the authors adjusted the spot size or pulse duration to deliver more energy to the area—such adjustments they stated could not be performed *in vivo* without damaging viable tissue—they could not reproduce the inhibitory effects on fungal growth.

Kalokasidis studied the Q-switched laser *in vivo* [5]. Out of 131 subjects with onychomycosis diagnosed by positive culture, he found 125 (95.4%) with negative fungal cultures at a three-month follow-up after being treated twice with the Q-clear Nd:YAG 1064/532 nm laser. Clinically, half the patients achieved at least a 50% reduction of diseased nail. For those patients with no clinical improvement, they suggested, “we do not have nonresponsive cases but some poor responding fungi”. The results were potentially confounded by mechanical debridement of the nails prior to treatment and limited by short follow-up time.

#### 3.1.4. Dual Wavelength Diode Laser

The Noveon is a dual-wavelength diode laser that emits light continuously at 870 and 930 nm through semiconductors rather than solid crystals. These wavelengths are preferentially absorbed by fungal cytochrome c oxidase and the lipid bilayer of the cell membrane, resulting in the generation of radical oxygen species causing photoinactivation of fungi [29]. Landsman and colleagues randomized 34 patients with culture-positive or PAS-positive onychomycosis of one or both great toes into a treatment group (25 patients, 26 toenails) and a control group with laser power set to zero (nine patients, 11 toenails), to receive four exposures to a 870/930-nm laser at days 1, 14, 42, and 120 [30,31]. At 180 days, a blinded expert panel found that 17 of the 26 treated toenails (65%) and one of 11 toenails in the control group (9%) attained at least 3 mm of clear linear nail growth (*p* = 0.011). Ten of 26 toenails in the treatment group (39%) and one of 11 in the control (9%) also demonstrated negative culture, however the difference with regard to culture was not statistically significant (*p* = 0.119).

On subjective assessment, the blinded expert panel found one toenail in the treatment group (4%) and two in the control (18%) to be markedly improved, *p*-values not reported. When assessing clearance of fungus by microscopy in eligible toenails as well as companion nails ineligible for the primary study, 64% were PAS-negative at day 120, diminishing to 46% at day 180. At 270 days, photographic assessment revealed 35% of all treated toenails with further improvement, 38% with no change, and 20% with worsening appearance. Patients were required to apply topical terbinafine and encouraged to debride nails regularly. Patients with onychomycosis extending beyond the eponychium or with luminal involvement were excluded from the trial. This study was funded exclusively by laser manufacturer Nomir Medical Technologies, and employees of the company were credited as authors of the paper. Both application of topical terbinafine and use of debridement were confounding factors, making interpretation of laser efficacy very difficult.

#### 3.1.5. Other Solid State Lasers

In addition to the Nd:YAG class, the category of solid state lasers includes titanium (Ti):sapphire as well as erbium based devices. With a pulse length in femtoseconds, the Ti:sapphire laser delivers a substantial amount of energy per pulse, but spares involved tissues of excessive heating because of a high thermal conductivity [32]. Manevitch and colleagues irradiated nail clippings from 99 patients with confirmed *Trichophyton rubrum* onychomycosis with an infrared Ti:Sapphire laser operated at an 800-nm wavelength and 200-fsec pulse duration [33]. The average power was adjusted so that the fluence varied from 10^31^ to 10^33^ photons m^−2^·s^−1^. The laser’s efficacy in eradicating the fungus was evaluated by subculture four weeks following treatment. The authors found that a minimum fluence of 7 × 10^31^ photons m^−2^·s^−1^ successfully inhibited growth of the fungus in 100% of samples, whereas laser intensities of 1.7 × 10^32^ photons m^−2^·s^−1^ affected the structure of the nail plate assessed by scanning electron microscopy.

Orlando de Morais and colleagues have found promising initial results for an erbium:YAG solid state laser [34]. They reported greater clearing rates of onychomycotic nails with a single session of 2940-nm Er:YAG laser in addition to application of amorolfine lacquer compared to those treated with amorolfine alone. The trial is ongoing and data are pending.

#### 3.1.6. Fractional CO_2_ Lasers

Indicated for matrixectomies, the fractional CO_2_ laser ablates the nail plate, potentially disrupting the growth of fungus as well [35]. Lim enrolled 24 patients with microscopy-confirmed onychomycosis to receive three sessions of a CO_2_ laser at four-week intervals, with instruction to apply topical amorolfine cream to the feet daily [9]. Efficacy was assessed by standardized photographs comparing the infected area at baseline and at 12 weeks. The authors found 12 patients (50%) with “complete cure”, defined as negative fungal culture in the setting of total visible clearance of infection, three months after the last treatment. The study was limited by small size and lack of a control, the application of topical amorolfine being a potential confounder. The authors did not remark on how many toenails in each patient were initially infected, and if multiple toenails, whether they cultured each nail to determine mycological cure.

### 3.2. Discussion

Lasers systems are increasingly presented as a convenient and effective panacea for onychomycosis. In light of the complications and extended treatment course associated with systemic antifungals, many physicians have embraced lasers as an alternative. However, on review of the data, a number of serious limitations are readily apparent. In addition to the lack of controlled experiments and small sample sizes enrolled in existing studies, much of the research to date is heterogeneous in design, with few sufficiently powered randomized controlled studies, making a conclusion of efficacy in laser treatment of onychomycosis difficult.

While many of the studies report a significant regression of onychomycosis with laser therapy, the lack of a standardized method for determining extent of infection poses a challenge for interpretation of the results. Across the board, a variety of laboratory tests were used to back assertions of onychomycosis cure. These included fungal culture, direct microscopy with potassium hydroxide, and periodic acid-Schiff staining, with respective sensitivities of 53%–59%, 48%–80%, and 88%–92% [36,37]. Although many authors relied on fungal cultures to diagnose and/or conclude eradication of infection, the sensitivity of this test is relatively poor compared to other modalities. It is estimated that nearly half of all specimens taken from onychomycotic nails fail to yield a positive culture [38]. In this context, a high rate of false negative cultures may have exaggerated mycological clearance. Although cultures are still considered the appropriate standard for determining the presence of infection post-treatment, their limitations are noteworthy when evaluating efficacy of any proposed therapy for onychomycosis. Similarly, inconsistencies in subjective cosmetic endpoints limit generalizability of conclusions.

Another limitation among current studies was the length of time between final treatment and follow up. Relapses or recurrences of onychomycosis are not uncommon, with percentages reported in various studies ranging from 10% to 53% [39]. Especially in cases with underlying *tinea pedis*, the question of whether cure as opposed to time-limited remission arises. Given these constraints, a premature conclusion of fungal clearance might well overlook persistent, subclinical infection responsible for recurrent clinical nail dystrophy after the transient effects of the laser have worn off. Although the ideal window for assessment has yet to be determined, the time required for toenails to fully grow out would be the earliest appropriate follow up. It takes approximately 12–18 months for a toenail to completely regrow, a time frame exceeding the vast majority of the study periods reported [29]. In a previous review of the literature, Bristow suggested a longer follow up of 24 months, given that nail growth occurs more slowly in those patients who are at highest risk for onychomycosis: diabetics, the elderly, and other immunosuppressed individuals [12]. Out of 16 studies, only two reported outcomes as far as 12 months beyond the final laser treatment. In both of these studies, efficacy was diminished at the final follow up with a significant portion of patients exhibiting relapse or recurrent infection. Given these outcomes, it is possible that many of the studies with a shorter window reported “success” prematurely.

Additionally, while lasers are commended for a lack of systemic side effects and drug interactions, there is little data investigating their safety. A primary concern is the nonspecific bulk heating that occurs when water in the tissue surrounding the nail absorbs laser energy, a phenomenon inherent to all Nd:YAG lasers [29]. An *in vivo* study showed that fibroblasts are damaged by exposure to 45 °C for twenty minutes, resulting in ulceration, dyspigmentation, and scar [40,41]. To place this figure in perspective, Carney argues that 50 °C is the lowest temperature able to achieve fungal wall lysis and death of *Trichophyton rubrum* [20]. If heat is taken to be the antifungal mechanism of lasers, this comparison suggests at minimum that effective temperatures would not be safely achievable. More extensive research is needed to identify whether effective temperatures may be safely reached without damaging the soft tissues or nail matrix.

## 4. Conclusions

In conclusion, most studies to date lack placebo or standard of care control groups, report different thresholds for significant clearance of debris from the nail, have limited enrollments, and have insufficiently interrogated safety profiles, hindering a meaningful conclusion of clinical benefit compared to previously existing options. In fact, per the premarket Section 5–510(k) Summaries submitted to the FDA, approval of each device has been sought for the limited indication of “temporary increase in clear nail” [42]. The evidence to date has not indicated superior outcomes in long-term end points to standard of care systemic therapy, and postulated anti-fungal mechanisms remain unverified.

Despite the scarcity of peer-reviewed literature investigating this topic, lasers have quickly risen to be considered among viable treatments for onychomycosis. As many offices have previously acquired a laser device for prior indications, it is understandable that physicians would be eager to discover additional innovative applications for these devices. Financial incentives may also have a role in widespread adoption. Sufficiently powered randomized control trials compared to previously existing therapeutic options are needed before lasers are deemed a standard of onychomycosis treatment. Moreover, head-to-head comparisons between different laser devices would be required in order to determine which device is optimum for the various morphological types of onychomycosis and the assorted etiologic fungi.

## Supplementary Files

Supplementary File 1
