# Supplementary material for: Laser Therapy for Onychomycosis: Fact or Fiction?
Source: J Fungi (Basel). 2015 Apr 3;1(1):44–54. doi: 10.3390/jof1010044 (PMC5770012; doi:10.3390/jof1010044)
Supplement: Supplementary File 1 [file jof-01-00044-s001.pdf]

Supplementary Information

Table S1. Laser Characteristics by Study.

Laser Model	Study	Model	Wavelength (nm)	Pulse Duration	Number of Treatments	Treatment Interval	Repetition Rate (Hz)	Fluence (J/cm^2)	Spot Size (mm)	Peer-Reviewed
**Short Pulse Nd:YAG**	Hochman (15)	Aerolase LightPod Neo	1064	0.65 ms	2–3	—	—	223	2	Yes
	Kimura (16)	Not specified	1064	0.3 ms	1–3	4, 8 weeks	5	14	5	Yes
	Waibel (17)	Sciton JOULE ClearSense *	1319, 1064, BroadBand	—	4	1 week	—	—	—	Yes
	Hollmig (18)	Sciton JOULE ClearSense *	1064	0.3 ms	2	2 weeks	6	5	6	Yes
	Choi (19)	Lutronic AccuSculpt	1444	0.1 ms	—	—	20	Total energy 150 J, 300 J, 450 J	—	Yes
	Carney (20)	Cutera Genesis Plus *	1064	*In vitro*, 0.1–0.3 ms	—	—	7–10	5–50	3–5	Yes
				In vivo, 0.3 ms	5	Weeks 0, 1, 2, 3, 7	2	16	5	
**Long Pulse Nd:YAG**	Zhang (22)	Nuvolase PinPointe FootLaser *	1064	30 ms	4, 8	1 week	1	240–324	3	Yes
	Moon (23)	Sciton JOULE ClearSense *	1064	0.3–200 ms	5	4 weeks	—	5	6	Yes
	Noguchi (24)	Candela GentleYAG	1064	0.5 ms	3	—	2	10	6	Yes
	Hees (25)	Nuvolase PinPointe FootLaser *	1064	0.1 ms	—	—	—	5–25	1–5	Yes
		Cynosure Elite	1064	40 ms	—	—	—	50	3	

Table S1. *Cont.*

Laser Model	Study	Model	Wavelength (nm)	Pulse Duration	Number of Treatments	Treatment Interval	Repetition Rate (Hz)	Fluence (J/cm^2)	Spot Size (mm)	Peer-Reviewed
**Q-switched Nd:YAG**	Hees (26)	Cynosure Affinity QS, Cynosure Elite	1064/532	6 ns, 40 ms	—	—	—	4–100	2–10	Yes
	Kalokasidis (5)	Light Age Q-Clear *	1064/532	9 ns	2	—	5	14	2.5	Yes
**Diode**	Landsman (30, 31)	Nomir Noveon *	870/930	—	4	Days 1, 14, 42, 120	—	204–424	1.5 cm	Yes
**Other Solid State Lasers**	Manevitch (33)	Coherent Mira 900 Ti:sapphire	800	200 fs	1	4 weeks	76 MHz	10(31)–10(32) photons $m^{-2}\cdot s^{-1}$	—	Yes
	De Morais (34)	Industra Etherea Er:YAG	2940	2 ms	—	—	1	—	—	No
**Fractional CO_2**	Lim (9)	Lutronic Mosaic eCO_2	—	—	3	4 weeks	—	—	—	Yes

* Approved in USA.

© 2015 by the authors; licensee MDPI, Basel, Switzerland. This article is an open access article distributed under the terms and conditions of the Creative Commons Attribution license (http://creativecommons.org/licenses/by/4.0/).
